# Conceptualisations of good care and conflicts in live-in migrant care arrangements for people with dementia – perspectives of family caregivers in Germany

**DOI:** 10.1186/s12877-024-05283-9

**Published:** 2024-08-24

**Authors:** Adele Grenz, Milena von Kutzleben

**Affiliations:** Division of Prevention and Rehabilitation Research, Carl von Ossietzky Universität Oldenburg, Ammerländer Heerstrasse 140, D - 26111 Oldenburg, Oldenburg, Germany

**Keywords:** Home care, Family caregivers, Dementia, Migrant live-in care, Autonomy, Quality of care, Good care, Conflict, Focus groups, Qualitative research

## Abstract

**Background:**

In Germany, live-in migrant carers provide essential social, emotional and physical support to a growing number of community-dwelling people with dementia. However, opaque legal regulations and employment models as well as a lack of formal supervision for families employing live-in migrant carers contribute to the vulnerability of these already strained arrangements. This study analyses the family caregivers’ perspective, their conceptualisations of good dementia live-in migrant care and conflicts that arise in live-in care arrangements.

**Methods:**

The study adopted a qualitative-explorative approach. We conducted focus groups with family caregivers (*n* = 15) to learn about their perspectives on and experiences with live-in care as a model of home-based dementia care. Due to the restrictions of the COVID-19 pandemic, data collection took place online, which enabled us to include participants from all over Germany in our sample. Data were analysed with qualitative content analysis.

**Results:**

In this paper, two main categories, *Indicators of good live-in migrant care for people with dementia* and *perceived conflicts*, are presented. We identified indicators applied by family caregivers to assess the quality of care provided by migrant live-in carers and its outcomes for the person with dementia. These relate primarily to interpersonal and emotional aspects and a person-centred attitude towards the person with dementia. Conflicts arise when the needs and personalities within the triad do not match, due to intransparent and unreliable work of and communication with the placement agencies, or permanent crisis as a result of the German model with alternating live-in carers.

**Conclusion:**

Our findings point to the complex dynamics and relationships within live-in care triads and support the theoretical assumption that taking into account the needs of all actors involved is essential for good and stable care arrangements. The conceptualisations of family caregivers of good dementia live-in migrant care offer starting points for a scientific as well as a social and health policy debate about the future regulation of this model of care.

## Introduction

Most of the almost 1.8 million people living with dementia in Germany [1] reside at home, usually cared for by the family alone or with the support of formal services [2]. Maintaining a stable and sustainable situation at home is a major challenge for informal caregivers. Most families try to avoid placement in a nursing home for as long as possible as they fear a loss of independence for the person with dementia and a loss of quality of care in the institutional setting [3]. In this situation, hiring a live-in carer from abroad seems to be a promising solution for many family caregivers [6, 7].

Live-in care is a common form of care in most high-income countries. Live-in migrant carers provide extensive (social) care, and they are often expected to be available 24-hours a day to ensure the elderly person’s safety and wellbeing and to relieve families from the burden of care. [9, 10].

In Germany, the live-in care model is not part of the formal long-term care system and follows the “migrant [caregiver] in the market” framework [11] with numerous placement agencies as competing actors [13]. The logic of this care market plays a significant role in determining the availability and competency of live-in carers and thus the quality of care [14]. Families may use in-cash benefits from the German social long-term care insurance scheme to hire a live-in carer. However, this has to be organised privately and additional out of pocket payments are necessary [15]. Live-in carers working in Germany mainly come from Eastern European countries and work in shuffle migration. Consequently, these live-in care arrangements are characterized by a frequent change of the live-in carer every few weeks [17, 18]. The legal framework for employing a live-in carer from abroad is opaque, and there are various models of employment. In most cases, families turn to one of the more than 400 placement agencies [13] for recruiting a migrant live-in carer, who is then either employed directly in the care household or deployed via a service contract between the care household and the agency. However, for families looking for a legally secure and fair care arrangement, the regulations and its consequences for live-in carers as well as for the family are hardly transparent. Moreover, there is a fundamental conflict in live-in care arrangements between the need for 24-hour care provided by one single person and good working conditions. Boundaries between working time, on-call time and leisure time of live-in carers become blurred and problems arise with regard to working hours and the German minimum wage regulations. As a consequence, live-in care often takes place in a legal gray area or illegality under labour or even criminal law [20, 21].

Dementia and its extensive care needs are often the reason for families to seek support by a migrant live-in carer. However, very few live-in carers received general training in care or even have a qualification in working with people with dementia. Caring for a person with medium or advanced dementia can be an emotional, physical and organizational challenge and there is great potential for conflicts if the ideas of good dementia care differ between the family and the live-in migrant carer, or if the live-in carer is overwhelmed by the dementia-specific care needs [23, 24]. In one of our own qualitative-reconstructive studies [22], it became apparent that family caregivers of people with dementia establish an individual informal care concept, which they, to a large extent implicitly, use to guide their care decisions and their care actions. These informal care concepts are determined by the definition of their own *role* as family caregivers, their *beliefs about dementia* and their *attitude towards the person with dementia*. From these informal care concepts, conceptualisations of good dementia care and expectations of external formal or semiformal support are derived. Conflicts in live-in care arrangements arise when the expectations of family caregivers are disappointed. Often, this disappointment results from contradictory (role) expectations. Live-in carers are expected to navigate the precarious balance between family closeness and professional distance, with role expectations ranging from family members to service providers to professional carers and moral actors [6].

Although live-in care is often used to meet the comprehensive care needs of persons with dementia, there is little empirical work to date that addresses this desideratum. For Germany, it is completely lacking.

### Aims and research interest

The aim of this study was to analyse the conceptualisations of family caregivers of good migrant live-in care for their relatives living with dementia. The focus of the analysis was set on the content of these conceptualisations and the conflicts that arise when the expectations attached to the conceptualisations are not fulfilled.

### Study design and methods

This study was framed by the cooperation project “Eastern European Live-In Carers in Domestic Care Triads in Dementia” (TriaDe) of the Division **of Prevention and Rehabilitation Research** and the Division **Ethics in Medicine at the Carl von Ossietsky Universität Oldenburg, Oldenburg/Germany**. In various qualitative-explorative sub-studies and systematic ethical analyses, live-in care provided by Eastern European caregivers as a model of domestic dementia care is investigated. In the subproject **TriaDe_online**, online focus groups were conducted with family caregivers of people with dementia on their views and experiences with live-in care.

### Sampling and data collection

Due to the difficulty of reaching the target group and the lack of registration of live-in migrant care arrangements in Germany, the recruitment strategy to reach out to family carers of people with dementia was conducted as broadly as possible. The call for participation was distributed electronically and in print as an information flyer via Alzheimer societies, dementia counselling centres, welfare organisations, the press service of our university and via the scientific networks of the authors. The inclusion criteria were: care-dependent family member has dementia, current, past or planned employment of a live-in carer from abroad, and sufficient German knowledge to participate in a focus group.

A total of 15 people meeting the inclusion criteria was included in the study (see Table 1).

**Table 1:** Sample characteristics (*n* = 15)

Characteristics of participants and circumstances of the live-in care arrangements		n*=
Gender	Female	10
	Male	5
Relationship to person with dementia	Adult child	11
	Partner	2
Duration of employment of live-in carers	One day to 30 days	2
	30 days to six month	2
	Six month to one year	3
	Longer than one year	5
Total number of live-in carers ever employed	One to two	2
	Two to five	5
	More than five live-in carers	4
Start of live-in care after onset of dementia	Less than one year	3
	One to three years-	4
	Longer than three years ago	4

* The information in this table comes from a short questionnaire. As not all participants completed the questionnaire in full, n does not always add up to 15.

The focus groups were conducted between July and September 2021. During this time, there were considerable contact restrictions in Germany due to the COVID-19 pandemic, so data collection had to take place online. The advantage of data collection online was that it allowed participants from all over Germany to be included and allowed low-threshold participation for people who could not travel far due to their care responsibilities or other reasons. We assumed that both participation in an online focus group and its facilitation required a high degree of concentration and flexibility, so the number of participants was set at three per focus group (n = five focus groups à three or two participants, respectively, plus one individual interview as two participants cancelled the planned appointment at short notice). Data collection was carried out using a conference tool (Webex by Cisco) approved by our institution for this purpose.

The focus groups were carried out as semi-structured interviews. The interview guide contained questions about the personal situation and experiences with live-in care, questions about the organization and negotiation of the care arrangement, and questions about live-in care in the special circumstances of dementia. Two researchers (**AD** and **MvK**, see acknowledgements) acted as moderator and protocolist during the discussions, which were audio recorded and transcribed verbatim. All data were pseudonymized prior to analysis. Data management and processing was carried out in compliance with the applicable data protection regulation, data will be stored for ten years.

### Data analysis

The data of the transcribed focus groups were analysed using qualitative content analysis according to Schreier [25] in the following steps: initial reading of the transcripts; inductive coding (see Table 1); development of main categories and assignment of subcategories; discussing the category system and adding inductive categories; final coding of all focus groups.

AG conducted the data analysis. The categories were developed in regular discussion loops between AG and MvK to check the plausibility and consistency of the categories. In case of dissent, the categories were discussed again until consensus could be reached. Table 2 presents an example of an analysis step.

**Table 2:** Example of an analysis step

Meaning unit selected from original text	Condensed meaning unit; Description close to the text	Interpretation of the underlying meaning	Developing a Subcategory	Assignment to main category
She [person with dementia] got along best with those who were very close to her, who also sought this emotional connection. … For her it was hard when someone kept a distance and said “I am the carer and that’s as far as it goes and no further”. (Group 4)	The person with dementia thrives with migrant live-in carers who seek emotional closeness, struggling when carers maintain an emotional distance.s	Emotional closeness and bonding promote the stability of the live-in care arrangement.	Allowing closeness and creating space for emotions	Indicators of good migrant live-in care for people with dementia

## Results

In the following, the two main categories with a total of seven subcategories of the content-analytical analysis of the focus groups are presented (see Table 3).

**Table 3:** Main categories and their subcategories

Main categories	Subcategories
Indicators of good live-in migrant care for people with dementia	Continuity of care in a safe and familiar environment
	Giving space to needs and preferences
	Preservation and promotion of resources and social participation
	Allowing closeness and creating space for emotions
Perceived conflicts	Lack of fit/ mismatching
	Intransparency and unreliability in the work of the placement agencies
	Instability and permanent crisis

### Indicators of good live-in migrant care for people with dementia

It turned out that family caregivers apply certain informal quality indicators to the care provided by live-in migrant carers, which are based on their individual conceptualisations of good dementia live-in care. Four subcategories could be identified.

### Continuity of care in a safe and familiar environment

For family caregivers, the quality of the arrangement is also measured by the assurance of continuity of care and the ability of the live-in carer to handle new or challenging phases as the dementia progresses. Objectively verifiable criteria, such as the physical condition, functionality and appearance of the person with dementia, are used as evidence of good and needs-oriented care and well-being of the person with dementia. These objective criteria are important for the family caregivers’ basic trust in the arrangement and in the live-in carer(s):

“At the end of the day, my mother is taken care of, she is happy, she laughs a lot, she is always happy when they are there [one of her live-in carers]. And that’s a reaction for me where I can say, okay, … it seems to work. She no longer loses weight, she walks around, and she has a healthy complexion. And yes, those are the criteria … because she can’t express whether or not she likes.” (Group 2).

The presence of a live-in carers averts health and safety risks for the time being. The desire for continuity of care is linked to the idea that live-in carers can classify dementia symptoms and deal with them in a person-centred way. A family caregiver who has established a sustainable long-term live-in care arrangement describes a shared learning process that opens up the possibility of understanding the dynamics of dementia:

“Because we had the last live-in carer for a very long time, almost six and a half years, they got to know each other very well, and that was a big advantage. When you find someone who can be there in that way…the carer can learn and grow along with the illness, so to speak”. (Group 6)

In addition, a known and reliable team of two to three alternating live-in carers ensures continuity of care. Family caregivers then experience that live-in care can maintain routines in the familiar environment. Respecting old habits makes it easier for the person with dementia and live-in carer to live together, reduces confusion and disorientation and promotes the autonomy of the person with dementia.

### Giving space to needs and preferences

A live-in carer who engages intensively with the person with dementia, learning about their preferences and needs, increases the quality of care and can thus provide the person with dementia with security and emotional stability. If live-in carers use the available time and peace of the home setting to adjust to the individual daily routine and behaviour of the person with dementia, they often succeed in creating a very needs-oriented relationship. Live-in carers and the person with dementia then make their own arrangements, which they negotiate independently and which are a perfect fit for these two actors in the triad:

“It was really about things like culture, rural conditions, religion. The things that were important to my parents. To get a lot of fresh air and also in terms of food, this, I’ll call it traditional, rustic, very meat-heavy food. So it was all quite wonderful. It was a good fit between my parents and the ladies [live-in migrant carers].” (Group 1).

### Preservation and promotion of resources and social participation

The cultural capital of live-in carers and their fit with the needs of the family being cared for favours the quality of interaction and communication and thus the quality of care. Linguistic competences and thus the ability to communicate verbally with the person with dementia are of great importance.

Family caregivers also experience a mixture of caring attention and activation as beneficial and describe the advantages when live-in carers bring different character traits and temperaments into the care arrangement:

“What’s important to me is that I feel that my mother is doing well. I think, with one of them, she enjoys being pampered, and with the other she is happy to have a bit of fire lit under her (laughs). So from that point of view, I think the mixture of the two is quite good.” (Group 2).

Quality is also measured by the organisation of shared activities. This includes meals but also walks or visits to cafés. Living with a live-in carer can give the person with dementia new incentives for mobility and sometimes also (re)enable social participation.

### Allowing closeness and creating space for emotions

Certain communicative skills of the live-in carers favour the quality and stability of the arrangement. The basis is an attitude of understanding, kindness, respect, recognition and closeness. Emotions are described as very important in dealing with the person with dementia, and relationships between the live-in and the person with dementia that are characterised by mutual closeness have a stabilising effect on the entire arrangement:

“She [person with dementia] got along best with those who were very close to her, who also sought this emotional connection. … For her it was hard when someone kept a distance and said “I am the carer and that’s as far as it goes and no further”. (Group 4)

If the chemistry is right, live-in carers and the person with dementia form a close bond, which is sensitively communicated within the framework of non-verbal and emotional signs and can defuse challenging behaviours such as aggression or agitation.

Family caregivers also attribute good care to the management of medication or the possibility of reducing it due to the situation being pacified by a live-in carer:

“I noticed when she spent a whole day in day care, she never got that attention. That’s when she got restless and started moving about a lot, that’s when she needed things to play with in her hands and stuff. And as soon as she came home, with the live-in carer, she was calm. We didn’t need any medication then either. In day care, we always needed medication to calm her down. And then she was still totally agitated when she came back home.” (Group 2).

### Perceived conflicts

The conceptualisations of good dementia live-in care give rise to conflicts when corresponding expectations are not met. The decision for live-in care is made in the face of a perceived lack of alternatives in view of the nursing home scenario and a lack of community-based structures and resources. It requires an adaptation process and negotiation process within the family to gain readiness to accept the risk of live-in care and a stranger in the home. In the course of this process, family caregivers intensively deal with their role, their possibilities and priorities. In this area of tension conflicts arise on a regular basis. Reasons and triggers for these conflicts are described below in three subcategories.

### Lack of fit/ mismatching

In general, the concept of live-in migrant care is evaluated by most family caregivers as a practicable way to enable the person with dementia to live a good life. However, the prerequisite for this is the individual fit:

“It doesn’t matter, we decided for this care option with an open mind and thought “We want to at least try it, see if it works”, and I think, possibly with a different person, it would have definitely worked.” (Group 2).

Conflicts often arise in arrangements when the attitude and interaction of the foreign live-in carer with the person with dementia do not match the ideas of the family caregivers. They then distance themselves from the care practices of live-in carers and dissociate themselves from their behaviour:

“They have a very different, a much harsher approach in some cases. So it’s a bit law and order. … let’s say, the basic attitude is different, it’s simply a different way of dealing with people, just a different culture.” (Group 5).

Often, the lack of fit is explained by perceived cultural differences. However, it is also experienced directly without its reasons and dynamics being understood. This is a problem specific to dementia because often the person with dementia cannot explicitly express his or her discomfort or is particularly dependent on implicit communication due to a lack of verbal communication options. Antipathies of the person with dementia towards the live-in carer, triggered, for example, by an unpleasant habit, a disturbing smell or a shrill voice, are experienced by family caregivers as a real danger to the success of the arrangement, which they are powerless against:

“You can’t get to the bottom of that, you can only acknowledge that it simply doesn’t work. And you really can’t do anything about it. It happens on a very fundamental level basically, a perception that you can’t influence.” (Group 5).

### Intransparency and unreliability in the work of placement agencies

In the focus groups, the discrepancy between the promises made by the agencies and the agreements made with the families and the experienced placement practice comes up repeatedly. The hope for the perfect match between live-in carer and person with dementia is too often disappointed; family caregivers feel patronized and perceive the allocation of live-in carer as “random”:

“It’s such a sham, really. Somehow, it’s like, “I’m sure they will like that [person]”. … What kind of live-in carer you eventually end up with is totally not what was discussed with the family in advance … so that already seems negligent or sometimes it felt like “Well, then they have someone, … and probably they’ll be happy.”… or just “I [the placement agent] know what they [the family] need” (Group 4).

The family caregivers observe a similar handling of the placement agencies with the live-in migrant carers, to whom, for example, the dementia is concealed.

Family caregivers often have very specific conceptualisations about what qualities and competencies the live-in migrant carers should bring, e.g., experience in dealing with dementia or solid knowledge of German. The placement agencies reinforce expectations in this regard by conveying to the families that they could virtually order a specific person with exactly these qualities. It is common practice for family caregivers to ask for specific desired competencies in advance by means of questionnaires or telephone calls, but the person sent then disappoints these expectations:

“And I think, why did I spent three hours filling out a form and talking to the guy five more times on the phone? Yes, and there’s always the question, is this a game of Telephone? Most of the time, you first talk to an agent in Germany, who makes the sales pitch. And then he passes it on to the agency that is located abroad. I don’t know if the information already gets lost there or if it is just that they simply say “I don’t really care.” (Group 4).

The disappointed expectations of family members as well as often lacking dementia-specific competencies and qualification of live-in migrant carers in combination with the explicit or implicit claim of many families for their 24-hour availability lead to conflicts and strain the arrangement.

### Instability and permanent crisis

Live-in arrangements in Germany are characterized by a regular change of live-in migrant carers due to the general conditions and the geographical proximity to the countries of origin. Typically, they rotate every 6 to a maximum of 12 weeks. Thus, the worry that the live-in carer might leave earlier, that the next person might not come, or that they might not fit in with the family, hovers constantly over the arrangement as a sword of Damocles. This fear of gaps in care puts a strain on family members, and the repetitive training of new live-in carers presents many of them with major challenges. This repetitive crisis situation can become a permanent burden on the entire family system:

“Then everyone broke out in panic. So the whole family is always freaking out when care is needed, but no one is there.” (Group 4).

Due to the lack of legal regulations for a complex demanding care arrangement, family caregivers feel overloaded, and the consequence often is exploitative employment. The impossibility of fair employment puts a strain on family caregivers and fundamentally calls into question the entire live-in arrangement:

“I couldn’t provide the required breaks. … even though I was there a lot, even though I took over a lot, even though I kept scheduling a lot of breaks. But I couldn’t manage these breaks required by law for a live-in carer. There was simply no way. And now, of course, if they add the fair wage thing.” (Group 2).

[This refers to a court decision on the claim of a live-in migrant carer for full remuneration of the hours actually worked. These were far above the regular weekly working hours of 40 h. On 24 June 2021, the Federal Labour Court ruled that a live-in carer is entitled to be paid for the full number of hours worked. The minimum wage applicable in Germany must be paid for the entire working time, including on-call times during the day and at night.]

## Discussion

The results of this qualitative-explorative study provide insight into the perspectives and experiences of family caregivers of a person with dementia concerning live-in care provided by migrant carers. To our knowledge, this is the first study in Germany that explicitly examined family caregiver’s perspectives on live-in care arrangements in the context of dementia and reveals their conceptualisations of good dementia live-in migrant care and experienced conflicts.

Live-in migrant care in Germany is barely regulated and state-controlled in terms of quality of care and there are no established standards. In contrast, the relatives’ perspectives analysed in the present study contained very specific ideas about what constitutes good live-in migrant care. In the live-in care model, which is governed by market laws, family caregivers play a central role in shaping the arrangement, negotiating care responsibilities and setting standards of good dementia care at the micro-level, as shown in our analysis. We were able to identify four indicators that are used to evaluate the live-in care arrangement and its accordance with the informal care concept [22] of the family caregiver(s). These include *continuity of care in a safe and familiar environment*, *giving space to needs and preferences*, *preservation and promotion of resources and social participation*, and *allowing closeness and creating space for emotions*. It becomes clear that the family caregivers’ ideas of what constitutes good live-in migrant care and expected outcomes relate primarily to the ability of live-in carers to provide person-centred care. The interpretation of the disease and the attitude towards the person with dementia are essential from the family caregivers’ perspective. Emotional relationships between the person with dementia and the live-in carer and meaningful interactions are valued as basic elements of good dementia live-in care. This prioritization of individual character traits and emotional and relational skills over professional training has been previously found in other studies on the perspectives of family caregivers [26, 27]. If one compares the informal perspectives in this study with regard to the ideas of good live-in dementia care with established concepts in dementia care [4, 5, 12, 16], fundamental similarities emerge. Concepts such as person-centeredness and the promotion of autonomy or social participation are important outcomes from the perspective of family caregivers in our study as well as in previous research [7, 19, 24, 28].

Nolan and colleagues [5] emphasise that the quality of care in the care of people with dementia depends above all on the care relationship and the ability of the carers to communicate and interact (relationship-centred care). In the case of live-in migrant care, this is a crucial aspect for family caregivers: they not only assume a management role that includes coordination tasks, e.g., agreements with the placement agencies, but they often function as mediators between the live-in migrant carer’s and the person with dementia’s needs [34, 35], and feel responsible for promoting good relationships within the live-in care arrangement. Leverton and colleagues [12] argue that dementia care should use the home of the person with dementia as an extension or expression of the self and integrate it into care (home-centred care). This idea is reflected in our results in terms of *Continuity of care in a safe and familiar environment* where family caregivers appreciate if live-in carers succeed in creating a needs-oriented care situation and a respectful and reliable relationship with the person with dementia.

Family caregiving in dementia means a trajectory of negotiating and decision-making within the family [22]. For the majority of our participants, the decision-making process around the live-in care arrangement turned out to be very challenging, both organizationally and emotionally. [7, 29]. According to the narratives of the family caregivers in our sample, conflicts arose when the conceptualisations of good live-in migrant care were not fulfilled [6]. As a consequence, the occurrence of conflicts leads to the entire concept of live-in care being called into question as a good and suitable solution for the family affected by dementia. Conflicts thus endanger the stability of the entire arrangement and often lead to manifest crises, when, for example, the live-in migrant carer is to be replaced immediately or the entire arrangement is terminated.

The participants in our sample were almost consistently negative about placement agencies. In particular, the loss of time and the futile effort to find suitable live-in migrant carers characterize disappointed expectations. The German model with alternating live-in migrant carers proves to be unfavourable from the families’ point of view, especially with regard to dementia. The repetitive change and fluctuation of live-in carers leads to a permanent emotional and organizational burden. Continuity of care and stable emotional relationships, especially with the person with dementia, are often hardly possible. Here, a dilemma inherent in this model of care becomes apparent: On the one hand, recovery times are indispensable, especially for live-in carers who are taking care of a person with dementia [30]; on the other hand, people with dementia are particularly dependent on relationship continuity. Live-in care arrangements must therefore be described as particularly vulnerable and fragile in the presence of dementia. These constellations are prone to conflicts and issues of trust as well as concerns about the quality of care are major challenges for families who decide to employ a live-in migrant caregiver [31, 32]. Although the family caregivers in our sample have had many challenging experiences, the model nevertheless seems to have the potential to enable people with dementia to live well in their own homes. However, the central condition for the success of a live-in arrangement is the fit with the informal care concept of the family caregivers and, above all, with their conceptualisations of good care in dementia.

At the moment, the social and academic debate on live-in migrant care is primarily focused on migration and (labour) law issues [33], structural inequalities and ethical aspects [34] of live-in migrant care [35]. In this context, dementia is often discussed as an aggravating factor. However, the discussion about good dementia care in the semi-formal context of live-in care is hardly ever held in public and has not been a priority in dementia-specific health services research. Without neglecting the legal and moral issues around live-in care in general, there is an urgent need for a discussion on how to conceptualize and improve dementia care within live-in care arrangements. It is essential to develop a legal framework that regulates organizational and financial aspects of live-in migrant care. This includes establishing standards for quality and safety of care and defining responsibilities of the state and social security policy, placement agencies and actors and live-in carers and families as actors on the micro-level. Furthermore, establishing education and counselling for families as well as for live-in migrant carers in order to make the model of live-in care more dementia friendly while addressing the needs and expectations of all actors involved.

### Limitations and methodological considerations

Our study has some methodological limitations. Due to the pandemic, there were additional challenges in recruiting a group that was difficult to identify and reach per se, as neither live-in migrant carers nor live-in arrangements are officially registered in Germany. In addition, family caregivers were even more burdened during the COVID-19 pandemic than they already were, which may have had a negative impact on willingness to participate in the study. Thus, we had to be satisfied with a convenience sample, and neither sociodemographic aspects nor the circumstances and constellation of the live-in arrangement could be taken into account. The online approach to data collection may have created an additional selection bias, as the participants had to have the willingness and the technical and personal resources to take part in an online focus group first. Due to the opaque framework conditions and the lack of legal regulation of and advice on live-in migrant care in Germany, a large number of illegal, abusive or failed arrangements can be assumed. Family caregivers with these experiences may be reluctant to participate in such research projects for fear of legal or moral consequences. In addition, the negative narratives may predominate because participants may have used the interviews to vent their frustration and negative experiences with live-in migrant care or placement agencies in particular.

Nevertheless, our analysis provides detailed insight into the lived experiences of family members of people with dementia in the context of live-in care. For the first time in Germany, the role of family caregivers in the micro-setting of domestic live-in arrangements is explicitly highlighted, and a detailed picture of their conceptualisations of good live-in migrant care and related conflicts can be reconstructed.

## Conclusion

This study’s findings highlight the challenges, gaps and potentials of live-in migrant care for people with dementia. From the perspective of health services research, the family caregivers’ perspective on live-in arrangements is of central importance because they are significantly involved in the organisation and maintenance of sustainable home care structures [8, 22]. However, our findings also point to the complex dynamics and relationships within live-in care triads and support the theoretical assumptions that taking into account the needs of all actors involved is essential for good and stable care arrangements [8]. For a comprehensive understanding of live-in migrant care in the context of dementia, the entire triad should be considered in future studies. Ethnographic approaches seem to be most promising in yielding insights into the everyday interaction in the of domesticity.

Live-in care arrangements are a care reality in Germany. However, this study highlights the complexity and fragility of live-in migrant care for people with dementia and once again points to the problematic framework conditions and their influence on the quality of care and the well-being of all actors involved. If this form of care is politically desired in the future, then, in addition to the question of fair employment conditions, a discussion of the question of the quality of care is unavoidable and persons with dementia are particularly vulnerable in this context. The conceptualisations of family caregivers of good dementia live-in care provided by migrant caregivers offer starting points for a scientific as well as a social and health policy debate about the future regulation of this model of care.

## Data Availability

The datasets generated and/or analysed during the current study are not publicly available due anonymity of the samples but are available from the corresponding author on reasonable request.
